# Small G Proteins Dexras1 and RHES and Their Role in Pathophysiological Processes

**DOI:** 10.1155/2014/308535

**Published:** 2014-03-20

**Authors:** Ashish Thapliyal, Rashmi Verma, Navin Kumar

**Affiliations:** Department of Biotechnology, Graphic Era University, Dehradun, Uttarakhand 248002, India

## Abstract

Dexras1 and RHES, monomeric G proteins, are members of small GTPase family that are involved in modulation of pathophysiological processes. Dexras1 and RHES levels are modulated by hormones and Dexras1 expression undergoes circadian fluctuations. Both these GTPases are capable of modulating calcium ion channels which in turn can potentially modulate neurosecretion/hormonal release. These two GTPases have been reported to prevent the aberrant cell growth and induce apoptosis in cell lines. Present review focuses on role of these two monomeric GTPases and summarizes their role in pathophysiological processes.

## 1. Introduction

Various physiological processes are synchronized by underlying molecular events and involve various signalling pathways. Our knowledge about molecular events involved in modulation of signalling pathways has increased immensely. Investigations involving role of monomeric G proteins in signalling pathways is still at its early stage. This review is an attempt to understand the role of two monomeric G proteins-Dexras1 (Dexamethasone-induced Ras-related protein 1) and RHES (Ras Homolog Enriched in Striatum) in modulation of various pathophysiological processes and justifying their importance as potential drug target.

**Figure 1:**
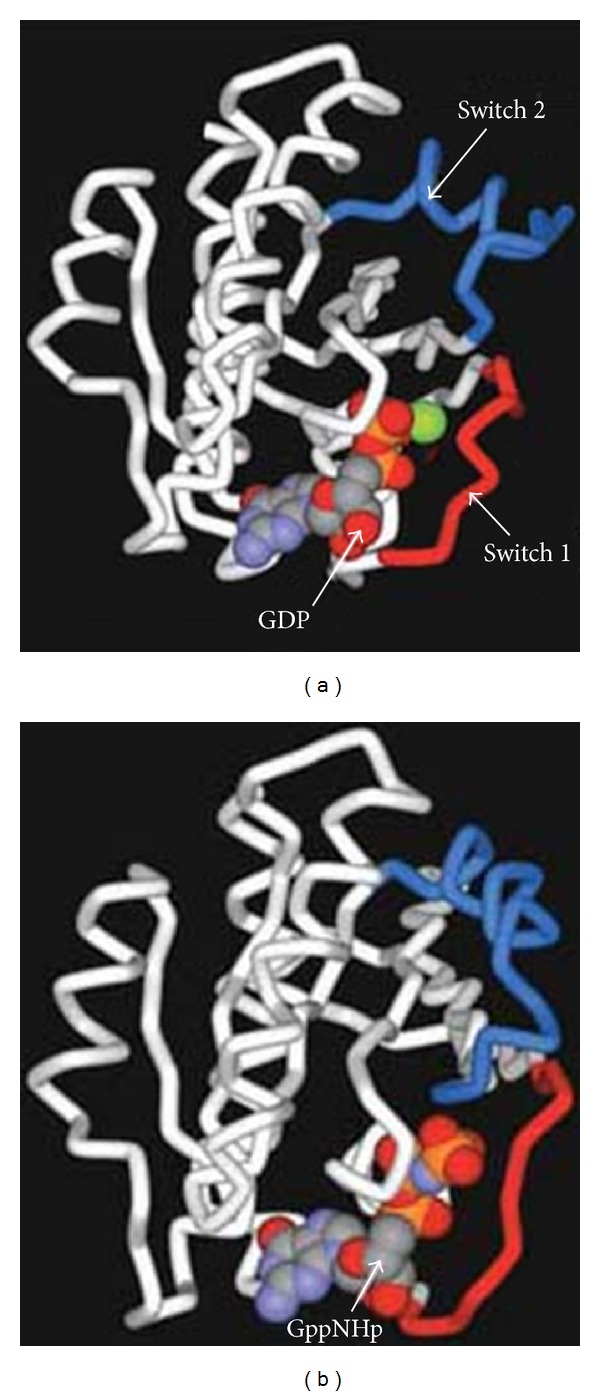
The switch regions of Ras. (a) Ras bound to GDP. This nucleotide is shown as a space-filling structure, with the magnesium ion (based on pdb file 4q21). (b) Ras bound to GppNHp (a nonhydrolysable analogue of GTP also shown as a space-filling structure). When accommodating the larger nucleotide, the switch 1 region appears to stretch and the switch 2 region swivels. (Based on pdb file 5p21. Reference: NCBI Protein Database).

Dexras1 and RHES both belong to RAS superfamily of small GTPase. Members of Ras superfamily are monomeric G protein, a guanosine-nucleotide-binding protein, which function as binary signalling switches with “on” and “off” states. Activation (on) and deactivation (off) of Ras and other small G proteins are controlled by cycling between the active GTP-bound and inactive GDP-bound forms [1] (Figure 1). Activation of Ras signalling triggers several pathways including those that cause cell growth, differentiation, and survival [1].

**Figure 2:**
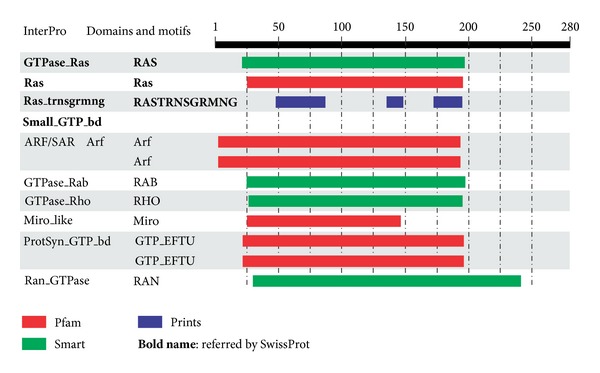
Different proteins that have similar domains like AGS1/RasD1/Dexras1. Dexras1 has been used as a reference protein in this figure and shows amino acids from 1–280. Reference: SwissProt.

There are many members of Ras subfamily like H-RAS, K-RAS, and N-RAS [2] and DIRAS1, DIRAS2, DIRAS3, ERAS, GEM, MRAS, NKIRAS1, NKIRAS2, NRAS, RALA, RALB, RAP1A, RAP1B, RAP2A, RAP2B, RAP2C, RASL10A, RASL10B, RASL11A, RASL11B, RASL12, REM1, REM2, RERG, RERGL, RRAD, RRAS, and RRAS2 (Figure 2) [3]. We are focusing on Dexras1 (RASD1) and RHES (RASD2). Dexras1 follows circadian pattern of expression in mice and level of expression of Dexras1 and RHES, both, is modulated by hormones (corticosteroids, estrogen, and thyroid hormones) [4–9]. Both these monomeric proteins are capable of modulating calcium ion-channels [10] which in turn regulate release of neurotransmitters in brain [7]. Their role in cardiovascular diseases, Huntington disease, and cancer has also been investigated [11–14].

## 2. Dexras1

Dexras1 is a protein that, in humans, is encoded by the* RASD1* gene. It is also known as* RASD1/AGS1* (activators of G-protein signalling 1). It belongs to the Ras superfamily of small GTPase [15]. Dexras1 was first discovered as a dexamethasone inducible monomeric Ras protein in At-T20 mouse corticotroph cells in the year 1998 and is expressed at high concentrations in brain and at lower concentrations in heart, liver, kidney, skeletal muscle, pancreas, and placenta [4, 5, 16, 17]. Expression of Dexras1 is upregulated by steroid hormones—Glucocorticoid, Dexamethasone, and *β*-estradiol. With reference to the mechanism involved in upregulation of Dexras1 expression by glucocorticoids, glucocorticoid response element (GRE) was identified in the 3′-flanking region (2.3 kb downstream of poly (A) signal) of the human Dexras1 gene. This element conferred rapid glucocorticoid responsiveness when inserted into a homologous promoter-driven luciferase reporter. This study suggested that the identified GRE is a key link to explain as to how Dexras1 gene responds to glucocorticoids with a rapid and profound induction [18]. Other stimuli reported to increase Dexras1 expression include desiccation stress, hypertonic stress, growth inhibitory stimuli (in B lymphocytes), and ischemia/reperfusion injury. It has been reported that, in mouse, Dexras1 expression is increased in the heart, brain, liver, and kidney in response to dexamethasone administration and in the pituitary in response to either *β*-estradiol or dexamethasone administration [4, 9, 19, 20]. However, the response elements for *β*-estradiol are yet to be identified.

**Figure 3:**
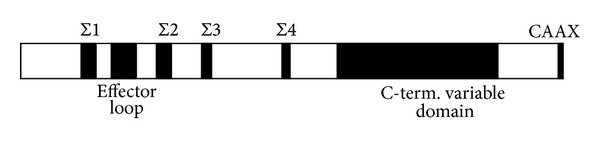
Schematic representation of the primary structure/motifs of Dexras1 contains all four components of the guanyl nucleotide binding and hydrolysis pocket (Σ1–Σ4) arranged with an order and spacing similar to that of other G proteins. An effector loop region similar to that of Ras family members and a carboxyl terminus CAAX box site for prenylation are evident. The residues spanning from the Σ4 domain to the CAAX box comprise an extended carboxyl terminus variable domain that accounts for the greater molecular mass of hormone-responsive, basic GTP-binding proteins as compared with other Ras family proteins. Reference: Graham et al. [9].

Dexras1 has all of the conserved domains of the Ras superfamily required for guanine nucleotide binding, hydrolysis, and effector interaction. The full-length cDNA of Dexras1 predicts a 280-aminoacid protein with a calculated molecular mass of 31,700 Da [4]. The structural organization of Dexras1 includes highly conserved GTP binding pocket (Σ1–  Σ4) domains and an effector loop which participates in protein-protein interactions with other signalling molecules and is necessary for full biological activity (Figure 3) [21–23]. The deduced structure of the Dexras1 protein contains several characteristic Ras superfamily motifs including the phosphate/magnesium binding regions G*XXXX*GK(S/T) (the P-loop), D*XX*G, and the guanine base binding loops NK*X*D and E*X*SAK [22, 24]. The motif regions G-1 through G-5 which are characteristic of GTPases are present in Dexras1 [22]. In addition, the C terminus has a typical CAA*X* motif [22, 24, 25], an important biochemical feature of a majority of Ras superfamily proteins. This CAAX motif undergoes enzymatic posttranslational modification (prenylation or farnesylation), which regulates its subcellular localization by promoting the translocation of the Dexras1 protein to the plasma membrane [3, 25, 26]. Prenylation is a type of lipid modification involving covalent addition of either farnesyl (15-carbon) or more commonly geranyl-geranyl (20-carbon) isoprenoids by thioether linkages to cysteine residues at or near the C terminus of intracellular proteins. The attached lipid is required for proper function of the modified protein, either as a mediator of membrane association or a determinant for specific protein-protein interactions. Prenylated proteins play crucial roles in such vital cellular processes as signal transduction and intracellular trafficking pathways [26]. These modifications are essential for facilitating membrane association and subcellular localization critical for biological activities [3].

Dexras1 may function as a guanine nucleotide exchange factor (GEF) for G*α*_*i/o*_ proteins [27] and, consequently, compete with G protein-coupled receptors to disrupt receptor-G protein signaling [28–30]. It has been reported that Dexras1 may have a dual role in modulating the activation of AC2 (Adenylyl cyclase 2) signaling by concurrently blocking PKC (protein kinase C) and G*βγ* activity—two proteins that function as activators of AC2. Dexras1 acts to negatively regulate PKC*δ* through an isoprenylation-dependent mechanism [31]. Dexras1 significantly reduced PKC*δ* autophosphorylation at serine 643 and the functional consequence was a loss of PKC*δ* catalytic activity. Dexras1 on PKC*δ* autoregulation is more likely to be a contributing factor toward its larger effects on AC2 activity. As Dexras1 can also regulate G*βγ* signaling [28, 30, 32] it may be that Dexras1 interferes with multiple inputs to AC2 that function in an additive or synergistic manner for maximal AC2 activity. The role for Dexras1 in regulating PKC*δ* activity may provide novel therapeutic targets for drug therapy, because many physiological and pathophysiological processes are associated with altered PKC*δ* signaling [31].

## 3. RHES

**Figure 4:**
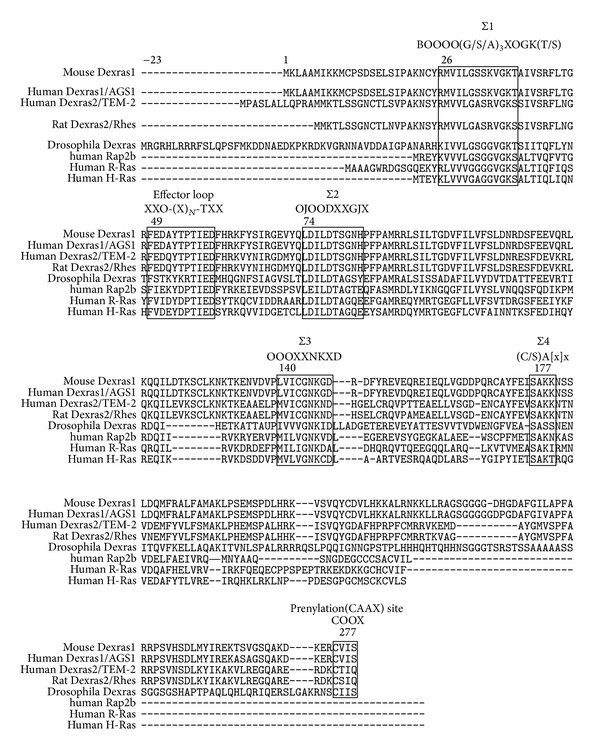
Alignment of Dexras1 with related hormone-responsive basic GTP-binding proteins (Dexras2, Drosophila Dexras) and representative Ras family members. Reference: Graham et al. [9].

RHES, also known as RASD2, is a novel striatal specific Ras-like small G protein exhibiting almost 62% similarity with Dexras1 (Figure 4 [33]). Expression of RHES is modulated by thyroid hormone [6]. It is assumed that thyroid hormones affect the normal development and functions of the brain by activating or suppressing several genes expressed in the brain [7]. Among these, the* RasD2* gene, encoding a small GTP-binding protein RHES, is predominantly expressed in the striatal region of the brain and is involved in striatal function [34]. RHES protein is expressed in different areas of the central nervous system such as striatum, olfactory tubercle, hippocampus (CA1, CA2, and CA3), cerebral cortex (parietal-layers 2, 3, 4, and 6), granular layer of cerebellum, and thalamus. However, its major level of expression is within the striatum and olfactory tubercle [8, 33]. RHES is also expressed outside of the nervous system in the thyroid and pancreas where it might regulate secretion of thyroid hormone and insulin, respectively [16, 35]. It is involved in selected striatal competencies mainly locomotor activity and motor coordination suggesting that its downregulation in hypothyroidism could be responsible only for a subset of symptoms, such as the striatopallidal syndrome typical of neurological cretinism [11, 36–38]. RHES is composed of 266 amino acids [4, 39]. Both RHES and Dexras1, as a distinct subclass, have an additional domain in the carboxyl terminal and N terminal region of the protein. These include an extended carboxyl terminus variable domain of about 56 amino acids in RHES (residues 210–266) and 70 amino acids in Dexras1 (residues 210–280) [33]. The N-terminal of both Dexras1 and RHES is also unique and differs from Ras family [9]. The C-terminal domain has been termed the “cationic region” as it is enriched in positively charged residues. The cationic region of RHES is essential for interaction with the G*β* subunits. RHES interacts specifically with the G*β*1, G*β*2, and G*β*3 subunits of heterotrimeric G proteins, but not G*β*4 or G*β*5 [40]. RHES is not an integral membrane protein but associates with the plasma membrane through posttranslational modifications on a CAAX domain [40].

## 4. Dexras1 and RHES Are Closely Related to Hormonal Levels

Expression of both proteins is under hormonal control. Dexras1 expression is induced by glucocorticoids like dexamethasone, corticosterone, and estradiol [4, 6, 9, 41]. It has been reported that the expression of Dexras1 is upregulated and increases in presence of dexamethasone and also in presence of corticosterone [41]. RHES is upregulated by thyroid hormones [5, 7, 8]. RHES is thyroid hormone dependent gene abundantly expressed in the caudate [6]. Hypothyroidism strongly decreases expression of RHES in the caudate region. T3 treatment normalized the expression of all genes. However, GC-1, a thyroid hormone analogue displaying selectivity for thyroid hormone receptor *β*, effectively normalizes expression of RHES and Reelin (thyroid hormone target genes) only [42]. It has also been reported that thyroid hormone modulation impacts striatal synaptic plasticity of adult mice that in turn might lead to motor behaviour modifications. Hypothyroid mice, treated with propylthiouracil (PTU) and methimazole (MMI) (antithyroid drugs inducing hypothyroid condition), have been used with or without subsequent administration of T3 [43] for experimental purposes. PTU and MMI (antithyroid) drugs are also used to treat hyperthyroidism. These drugs decrease the amount of thyroid hormone produced by the thyroid gland by inhibiting the enzyme thyroperoxidase. After inducing hypothyroid condition in mice with the antithyroid drugs, the variations in amount of proteins involved in striatal synaptic plasticity and motor behaviour have been evaluated. These proteins include T3 nuclear receptors (TR*α*1, TR*β*), neurogranin (RC3), Ras homolog enriched in striatum (RHES), Ca2+/calmodulin-dependent protein kinase (CaMKII), and dopamine- and cAMP-regulated phosphoprotein (DARPP-32). Hypothyroid mice exhibited significantly reduced TR*β*, RC3, and RHES expression. It has been observed that T3 administration reversed the expression of TR*β* and RC3 and upregulated CaMKII levels as well as motor behaviour and decreased DARPP-32 protein phosphorylation. These findings suggest that T3 administration in adult hypothyroid mice modulates expression of proteins involved in striatal synaptic plasticity and improves motor behavior [43].

It has been reported that Dexras1 and RHES also influence the secretion of other hormones. Dexras1 plays a key role in growth hormone (GH) regulation. It significantly inhibited CORT (corticosterone) induced GH expression and at lower doses may stimulate basal GH expression [44]. It has been suggested also that regulation of Dexras1 (only in presence of glucocorticoids and prolactin) controls peripartum maternal insulin secretion. The transition from gestation to lactation is characterized by a robust adaptation of maternal pancreatic *β*-cells. Consistent with the loss of *β*-cell mass, glucose-induced insulin secretion is downregulated in the islets of early lactating dams. Dexras1 is localized within pancreatic *β*-cells. Its expression in insulin-secreting cells was increased by dexamethasone and decreased by prolactin [41]. Knockdown of Dexras1 abolished the inhibitory effects of dexamethasone on insulin secretion and the protein kinase A, protein kinase C, and ERK1/2 pathways. The stimulation of Dexras1 expression by glucocorticoid at the end of pregnancy reverses the increased insulin secretion that occurs during pregnancy. Prolactin negatively regulates this pathway by inhibiting GR/STAT5b transcriptional activity on the Dexras1 gene [41].

## 5. Role of Dexras1 and RHES in Various Pathophysiological Processes

### 5.1. Role in Cardiovascular Diseases

In recent studies, Dexras1 showed its therapeutic implications for cardiovascular diseases. Atrial natriuretic factor (ANF) is a powerful vasodilator and a protein secreted by heart muscle cells. It is involved in homeostatic control of body water, sodium-potassium, and fat. It is released in muscle cells in the upper chamber (atria) of the heart in response to high blood pressure. It binds to specific receptors and causes reduction in blood volume and therefore reduction in cardiac output and systemic blood pressure. The overall effect of ANF is to counter increase in blood pressure. In volume overload (VO) condition of atria, significant downregulation of Dexras1 has been reported.* In vitro*, knockdown of Dexras1 in the atrial-derived HL-1 cells is reported to increase ANF secretionsignificantly. Concurrent knockdown of Dexras1 and its effectors G*α*(o1) or G*β*(1)*γ*(2) reduced the endocrine response, demonstrating a previously unknown negative modulator role for Dexras1. Thus, Dexras1 is emerging as a tonic inhibitor of ANF secretion and acts as a modulator of hormone secretion in volume overload condition of heart by inhibiting protein regulation of ANF release. Thus, there might be a novel molecular function and therapeutic implications of Dexras1 in cardiovascular disease [12].

### 5.2. Role of RHES in Huntington Disease

Huntington's disease (HD) is an inherited neurological disorder that causes a wide range of symptoms including involuntary movements, clumsiness, lack of concentration, memory lapses, mood swings, and depression. It is caused by an abnormal expansion of a CAG repeat located in exon 1 of the gene encoding the huntingtin protein (Htt). Abnormal huntingtin proteins or mutant huntingtin protein (mHtt) aggregates and forms clumps. There are fewer of these clumps in the corpus striatum of HD patients than in other brain regions or elsewhere in the body suggesting that clumping of the protein may actually somehow protect the cells. Addition of RHES to cells with abnormal huntingtin protein (mHtt) led to fewer clumps suggesting that RHES might be responsible for preventing abnormal protein from clumping. RHES does prevent clumping but it has been demonstrated that the cytotoxicity of mutant Htt is greatly enhanced in the presence of RHES protein. The HD patient shows selective atrophy of the striatum. RHES, expressed in striatum, was found to bind much more tightly to mutant huntingtin than to normal protein. RHES modifies mHtt through sumoylation, a posttranscriptional process that consists of the addition of the protein SUMO1 (small ubiquitin like modifier) to mutant Htt (mHtt). RHES has the properties of a SUMO-E3 ligase and mediates mutant huntingtin (mHtt) cytotoxicity [45]. The RHES-mediated sumoylation of mutant Htt eventually leads to its disaggregation and augmented neurotoxicity by increasing level of the toxic soluble form of mutant Htt. These findings lead to new therapeutic strategies to design drugs which will specifically target RHES to treat HD as it expresses in striatum and mediates mutant Htt toxicity [13, 46, 47].

### 5.3. Role in Regulation of Rhythms

It has been suggested that Dexras1 regulates the circadian clock. It undergoes a circadian pattern of expression and is implicated in modulating photic and nonphotic responsiveness of the circadian clock [39, 48, 49]. The mammalian master clock, located in the suprachiasmatic nucleus (SCN) [50], is exquisitely sensitive to photic timing cues and Dexras1 is a critical factor in these processes. It is suggested that synchronization of circadian cycles or circannual cycles (biological clock) with the environment is achieved mainly due to entrainment by light-dark (LD) cycle or a critical period of day length increase might synchronize biological clock to environmental cycle [51]. Other secondary factors might also entrain a biological clock, for example, food availability [52].

Dexras1 plays an important role in regulating the behavioral outcome of temporal restricted feeding and the response of the SCN to RF. It has been reported that loss of Dexras1 has a profound effect on light-entrainable rhythms and timing of the SCN clock. The study said that scheduled feeding alters the timing of the suprachiasmatic nucleus circadian clock in Dexras1-deficient mice [53]. Animals adapt to conditions of limited food availability by increasing food-seeking behavior, or FAA (food anticipatory activity), in the hours preceding food presentation. Role of Dexras1, a modulator of multiple inputs to the SCN (the central circadian pacemaker in mammals), in regulating the effects of Restricted feeding (RF) on activity rhythms and gene expression in the SCN has been examined. Circadian rhythms of FAA are thought to be controlled by a food-entrainable oscillator (FEO) outside of the suprachiasmatic nucleus (SCN); RF schedules are potent zeitgebers capable of entraining metabolic and hormonal rhythms in peripheral oscillators in anticipation of food. The augmented expression of FAA in the Dexras1 deficient mice may, therefore, be explained by greater reduction in the suppressive effects of the SCN on FEO outputs under food-restricted conditions. This may be due to loss of Dexras1 expression within the SCN, itself, or alternatively within the FEO (independent of any effects of the SCN). This study reported that genetic ablation of Dexras1 heightens the sensitivity of SCN-driven rhythms to the synchronizing effects of daytime RF. Thus, it was found that the absence of Dexras1 sensitizes the SCN to perturbations resulting from restricted feeding [53].

Circadian clocks synchronize the physiology and behaviour of most animals with the day to night cycle. Specific groups of circadian neurons have dedicated function in the control of circadian behaviour and its responses to temperature and light inputs [54]. Flies and mammals both rely on dedicated circadian photoreceptors (CRY, melanopsin) and on canonical visual photoreception to synchronize circadian rhythms with the LD cycle [55–57]. There are many proposed theories about entrainment of biological clock. It is also suggested that most of the regulators of biological clock (per, cry genes/gene products) undergo posttranslational modification especially phosphorylation by casein kinase I (CK-I) and Dexras1 has been shown to have phosphorylation site for CK-I [58].

**Figure 5:**
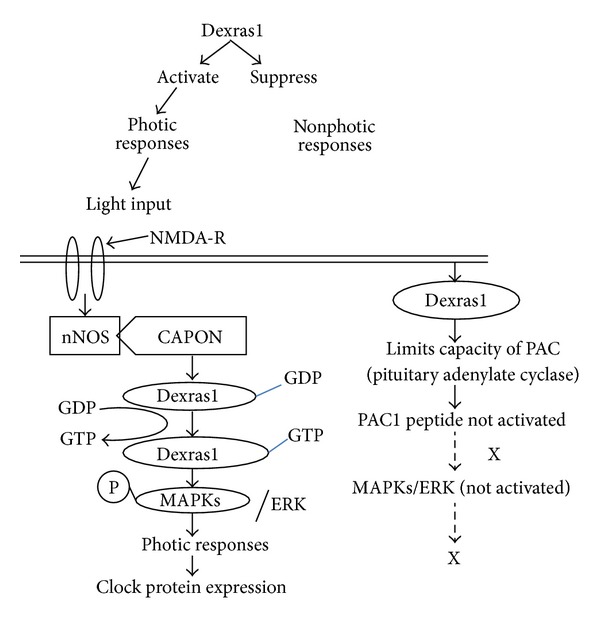
Dexras1 activates photic response and suppresses nonphotic response. Dexras1 activate the photic response by signalling pathways that regulate extracellular signal-regulated kinase (ERK)/mitogen-activated protein kinase (MAPK). The ternary complex (nNOS, CAPON, and Dexras1 bound to GDP) receives an external signal of light through NMDA receptor and leads to S-nitrosylation of Dexras1 on cysteine 11, an apparent prerequisite for GTP binding by which it becomes active to produce downstream signalling and produce photic response. In absence of external signal, light input, Dexras1 suppresses the nonphotic response by limiting the capacity of Pituitary Adenylate Cyclase (PAC so PAC1) to get activated; hence MAPKs/ERK is not activated and photic response not produced.

**Figure 6:**
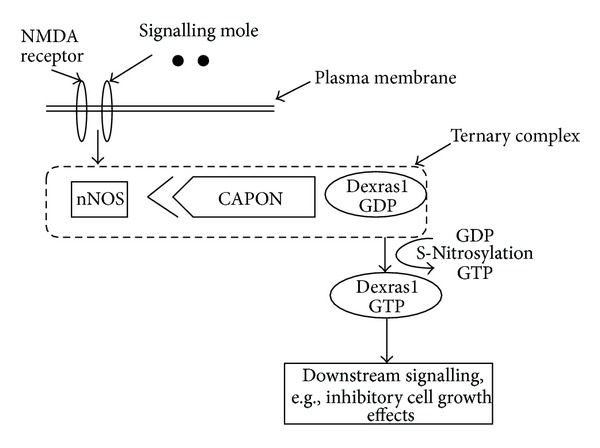
Dexras1 bound to GDP, nNOS, and CAPON can exist together as a ternary complex. NMDA receptors activation leads to S-Nitrosylation of Dexras1. S-Nitrosylation of Dexras1 leads to exchange of GDP with GTP and this active Dexras1-GTP initiates downstream signals.

Dexras1 has also been shown to undergo circadian variation in its expression and activity in mice but its role in modulation of circadian rhythms is still being debated [39, 48–51, 58–60]. Dexras1 has been shown to be a downstream physiologic target of neuronal nitric oxide synthase (nNOS)-mediated signalling [61, 62] regulating both photic and nonphotic input into the circadian clock in the suprachiasmatic nucleus (SCN). This signalling cascade requires coordination of NMDA receptor signalling, GPCR signalling, and ERK1/2 activation. In vivo studies reported that mice lacking Dexras1 expression exhibit altered regulation of both photic and nonphotic responses in the mammalian circadian clock. Dexras1 affects the photic sensitivity by repressing or activating time-of-day-specific signalling pathways that regulate extracellular signal-regulated kinase (ERK)/mitogen-activated protein kinase (MAPK) [20, 63]. Dexras1 has been implicated as a receptor-independent activator of Gi/o-protein signaling [64] as well as a context-dependent modulator of the MAPK cascade and other signal transduction pathways, including adenylyl cyclases (ACs) and NMDA receptor-nitric oxide- (NO-) mediated signaling [19, 29, 32]. In the early night, light-induced activation of NMDA receptors leads to a nitrosylation-dependent enhancement of the guanine nucleotide exchange activity of Dexras1 by which Dexras1 activates the MAPK pathway and promotes photic resetting. Light exposure in the late night leads to activation of Gs-coupled PAC1 (Pituitary Adenylate Cyclase1) receptors, which signal via both the G*α*s and G*βγ* limbs to the MAPK cascade. Dexras1 limits the capacity of Pituitary Adenylate Cyclase (PAC) and inhibits PAC1-mediated MAPK pathway activation by suppressing G*βγ* signaling events as well as AC (adenylyl cyclase). Dexras1 may inhibit AC indirectly by a receptor-independent enhancement of tonic G_*i/o*_ *α* activity. (Figure 5). The extended 7 kDa C-terminal cationic domain of Dexras1 was identified as a binding partner for the C-terminal PSD95/DLG/ZO-1 ligand of nNOS (CAPON) [64], a scaffolding protein that interacted with nNOS [65] and forms a ternary complex. When the ternary complex (bound to GDP) receives an external signal through NMDA receptor, it leads to S-nitrosylation of Dexras1 on cysteine 11, an apparent prerequisite for GTP binding by which it becomes active to produce downstream signalling [19] (Figure 6). In a recent study NonO is identified as a binding partner of Dexras1 [66]. NonO is a member of the family of RNA-Recognition Motif (RRM) containing proteins [67]. It is a coactivator of CREB (cAMP response element- binding protein) and has been known to serve in both transcriptional activation and repression [68–71]. In the nucleus, Rasd1 binds to NonO and regulates the cAMP-dependent pathway at the transcriptional level. Binding of Rasd1 to NonO modulates NonO's functions by changing NonO from a coactivator to a corepressor of the cAMP dependent pathway which is associated with the repression of a subset of CREB target genes, NR4A1 and NR4A2. This process involves the GTP hydrolysis activity of Rasd1 and requires interaction of Rasd1 with full-length NonO at the CRE-site of the target promoter. NR4A1 and NR4A2 are clock-controlled genes oscillating in multiple tissues [72, 73] whose expressions are upregulated upon activation of the cAMP pathway [69]. Hence, modulation of NR4A1 and NR4A2 expression by Rasd1 and NonO could have a major impact on the circadian control, and disruption of this process can give rise to metabolic diseases and cancer development [72–75]. Thus, Dexras1 becomes an important protein that might modulate biological rhythms.

### 5.4. Role of RHES/Dexras1 in Neurotransmitter Mediated Behaviour(s)

RHES affects Dopamine (D1 and D2) receptor mediated behaviour(s). Investigation reported that, in mice, RHES is normally inhibitory to behaviours induced by D1/D2 receptor costimulation and by D2 receptor stimulation alone. However, RHES appears to facilitate the D1-specific behaviour of grooming [76]. It has also been reported that RHES protein levels affect locomotion activity and have an influence on anxiety depending on the gender but RHES protein levels do not affect D1/D2 synergism in both genders [77]. It has been suggested that RHES and Dexras1 affect signalling by dopamine D1 receptors through adenylyl cyclase [78]. It has also been reported that amphetamine (AMPH, a psychostimulants) upregulates Dexras1 expression in the prefrontal cortex (PFC) of rat. The effects of AMPH on Dexras1 expression in the PFC, blocked by a D2 (dopamine receptor Antagonist) and partially by a glucocorticoid receptor antagonist, parallels behavioural activation by acute AMPH in drug-naive animals and hypersensitivity to AMPH challenge in sensitized animals. Changes in Dexras1 levels in the PFC might result in abnormal receptor to G protein coupling that alters cortical sensitivity to psychostimulants [79]. Besides this, changes in expression pattern of Dexras1 by alcohol exposure have been reported in case of prenatal embryos. Levels of Dexras1 were downregulated in embryos when mother mice were exposed to alcohol. Hence, both these GTPases can be potential therapeutic targets in case of substance of abuse [80].

### 5.5. Role in Cancers

Role of Dexras1 has also been suggested in cancer (an aberrant and uncontrolled growth of cells). Dexras1 is the member of RAS superfamily which belongs to cell growth, differentiation, and survival, but an investigation has also reported that Dexras1 suppresses aberrant cell growth. In clonogenic assays with NIH-3T3 murine fibroblast cells, the MCF-7 human breast cancer cell line, and the human lung adenocarcinoma cell line A549, Dexras1 transfection markedly diminished the number of G418-resistant colonies, whereas K-Ras, another member of the Ras protein family, was without effect. A549 cell infection with adenovirus engineered to express Dexras1 inhibited log phase growth* in vitro* and increased the percentage of cells undergoing apoptosis. The antigrowth action was also observed* in vivo* as the expression of Dexras1 inhibited the subcutaneous tumour growth of A549 cells in athymic nude mice. These data indicate that Dexras1, as a member of RAS superfamily, often promotes normal cell growth, but also plays an active role in preventing aberrant cell growth (cancer) [14]. Recently it has been reported that Calycosin induces apoptosis by upregulation of Dexras1 in human breast cancer cells MCF-7. Calycosin, one of the main components extracted from Chinese medical herb* Radix astragali*, at low concentration stimulated proliferation of ER-positive MCF-7 human breast cancer cells. High concentrations of calycosin significantly suppressed the proliferation of MCF-7 cells and promoted cell apoptosis. The expression of Bcl-2 (an antiapoptosis protein that inhibits or suppresses apoptosis) decreased with calycosin in MCF-7 cells and the expression of Bax (a protein that accelerates programmed cell death by binding to, and antagonizing, the apoptosis repressor Bcl-2) increased, which was significantly correlated with elevated expression of Dexras1. Dexras1 is a regulator in MAPK-mediated cascade leading to cell proliferation or apoptosis. These observations suggested that relatively high concentration of calycosin triggered cell apoptosis through the mitochondrial apoptotic pathway by upregulating Dexras1 [81]. Thus, Dexras1 might also be an important therapeutic target for cancer.

## 6. Future Perspectives

In present times, as the life style changes, populations in general are under a lot of stress due to their work, maintaining family survival, and their needs. Stressful lifestyle and situations are possibly related to many pathophysiological conditions. This review has focused on two GTPases, Dexras1 and RHES, which alters the hormonal level in the body and the expression of these two GTPases is regulated by hormones which get altered in stress condition. These two GTPases have role in many pathophysiological processes like Huntington disease, cardiovascular disease, regulation of rhythms, neurotransmitter mediated behaviour, and cancer. Focusing on these two GTPases might result in potentially treating many pathophysiological conditions and stress related diseases. Hence the present review suggested that Dexras1 and RHES can be the potential therapeutic targets of immense importance in future.
